# Chemokine CXCL12 drives pericyte accumulation and airway remodeling in allergic airway disease

**DOI:** 10.1186/s12931-022-02108-4

**Published:** 2022-07-13

**Authors:** Rebecca Bignold, Bushra Shammout, Jessica E. Rowley, Mariaelena Repici, John Simms, Jill R. Johnson

**Affiliations:** 1 School of Biosciences, College of Health and Life Sciences, Aston University, Birmingham, B4 7ET UK; 2 Department of Life Sciences, Faculty of Natural Sciences, Imperial College London, London, SW7 2AZ UK

**Keywords:** Allergic airway disease, Airway remodeling, Pericyte, CXCR4, LIT-927

## Abstract

**Background:**

Airway remodeling is a significant contributor to impaired lung function in chronic allergic airway disease. Currently, no therapy exists that is capable of targeting these structural changes and the consequent loss of function. In the context of chronic allergic inflammation, pericytes have been shown to uncouple from the pulmonary microvasculature, migrate to areas of inflammation, and significantly contribute to airway wall remodeling and lung dysfunction. This study aimed to elucidate the mechanism by which pulmonary pericytes accumulate in the airway wall in a model of chronic allergic airway inflammation.

**Methods:**

Mice were subjected to a protocol of chronic airway inflammation driven by the common environmental aeroallergen house dust mite. Phenotypic changes to lung pericytes were assessed by flow cytometry and immunostaining, and the functional capacity of these cells was evaluated using in vitro migration assays. The molecular mechanisms driving these processes were targeted pharmacologically in vivo and in vitro.

**Results:**

Pericytes demonstrated increased CXCR4 expression in response to chronic allergic inflammation and migrated more readily to its cognate chemokine, CXCL12. This increase in migratory capacity was accompanied by pericyte accumulation in the airway wall, increased smooth muscle thickness, and symptoms of respiratory distress. Pericyte uncoupling from pulmonary vessels and subsequent migration to the airway wall were abrogated following topical treatment with the CXCL12 neutraligand LIT-927.

**Conclusion:**

These results provide new insight into the role of the CXCL12/CXCR4 signaling axis in promoting pulmonary pericyte accumulation and airway remodeling and validate a novel target to address tissue remodeling associated with chronic inflammation.

## Introduction

Allergic asthma is an incredibly common and widely researched disease, with over 5.4 million people suffering from asthma in the UK [1] and 339 million people worldwide [2]. Asthma is characterized by the presence of airway remodeling, such as increased smooth muscle accumulation around the airways, collagen accumulation within the airway wall, excess mucus production and epithelial shedding as well as angiogenesis [3]. These structural changes cause the airway wall to thicken and narrow; consequently, airflow is decreased, and lung function is impaired [4]. This results in the characteristic symptoms seen in asthmatics, including episodes of wheezing and dyspnea [5]. Several factors contribute to this remodeling process, including inflammation and associated fibrotic mediators such as TGFβ.

Current treatments for asthma involve either symptom relief using bronchodilators or anti-inflammatory corticosteroids [6]. Despite the effectiveness of these therapies, studies have identified that more than half of patients with the disease have uncontrolled asthma [7]. Currently, no therapies exist for the treatment of fibrosis, despite it being a distinctive feature of many chronic inflammatory diseases. However, much research has been carried out to identify the cellular origins of fibroblasts in fibrotic organs [8, 9]. Previous work has identified a perivascular origin of myofibroblasts contributing to fibrosis in the kidney and the liver [8, 9], and we have identified the involvement of pericytes in driving airway remodeling in a mouse model of allergic lung disease, where pericytes were found to accumulate in the airway wall in the context of chronic aeroallergen exposure [10]. Pericytes are a heterogeneous population of perivascular cells that surround microvessels [11]. Typically, pericytes demonstrate an elongated stellate morphology with a cell body, nuclear region and numerous processes that surround the vessel. However, due to their heterogeneous nature, the morphology and function of pericytes vary greatly and depend on factors such as the resident tissue and signals from the local environment [11–13]. Pericyte functions include, but are not limited to, vessel stabilization, microvasculature homeostasis as well as vascular development and angiogenesis [14].

The role of pericytes is allergic asthma is linked to their contributions to airway remodeling, in particular myofibroblast differentiation and airway smooth muscle thickening [10]. A key factor in this process is the uncoupling of pericytes from their resident blood vessels and migration towards the inflamed airway, driven by impaired PDGFRβ signaling in pericytes in response to chronic allergic inflammation [10]. However, little is currently known of the migratory capacity of pericytes. Bone marrow mesenchymal stem cells (MSC), which have many characteristics in common with tissue pericytes, have been shown to express the receptor CXCR4, and the CXCR4/CXCL12 axis is thought to be the means by which these cells are retained within the bone marrow [15]. CXCL12 has also been shown to be upregulated during tissue injury, and while the dynamics of this upregulation are disputed, research has shown that the blockade of CXCR4 results in reduced MSC recruitment following burn injury [13, 16]. However, other data suggest that binding of the CXCR4 antagonist AMD3100 serves to increase the mobility of MSCs and enhance their recruitment to bone fractures in mice [17]. The relevance of the CXCR4/CXCL12 axis in pericyte recruitment and migration during tissue injury/inflammation is yet to be determined.

Building on previous work showing that pericytes uncouple from the vasculature and migrate to the inflamed airway wall and thereby contribute to airway remodeling, this study aimed to elucidate the means by which pericytes migrate to sites of inflammation and fibrosis in the lung. To this end, we assessed the migratory capacity of these cells ex vivo and in vitro using Transwell assays to assess pericyte migration. We furthermore assessed chemokine receptor expression in pericytes derived from normal mouse lungs and from the lungs of mice subjected to allergen-driven pulmonary inflammation and fibrosis. Under these fibrotic conditions, characterized by high levels of CXCL12 expression in the airways, pericytes were observed to uncouple from the airway microvasculature and demonstrated enhanced migration toward chemotactic stimuli. Finally, using a novel neutraligand to suppress CXCL12 activity (LIT-927) [18], we were able to mitigate pericyte uncoupling from the airway microvasculature, resulting in decreased airway smooth muscle accumulation and improved symptom scores.

## Methods

### Human pericyte culture

HPLPCs (human placental pericytes cells) from Promocell (Heidelberg, Germany) were cultured for use in experimentation. They were received from Promocell at passage 2 and cultured until passage 10 as beyond this they began to display an altered phenotype. All pericytes were cultured in specific pericyte growth media (Promocell, Heidelberg, Germany) with additional 1% antibiotic–antimycotic (ThermoFisher, Massachusetts, US). They were cultured in flasks and well plates coated in a gelatin mixture of autoclaved distilled water with 5% gelatin to ensure proper attachment of cells. Trypsin–EDTA (Sigma-Aldrich Ltd., St. Louis, USA) was using during passaging to allow cells to detach from the culture surface.

### In vivo house dust mite model

All animal procedures were carried out in strict accordance with the approved protocol and recommendations for proper use and care of laboratory animals (Animals (Scientific Procedures) Act 1986). All experiments on animals were conducted according to United Kingdom Home Office regulations (project license P75A73BEB held by the corresponding author) and animal handling was performed by qualified personnel. All studies were performed and reported according to the revised ARRIVE guidelines [19].

Thirty female C57Bl/6 mice (6–8 weeks old) were purchased from Charles River and housed at the Imperial College London central animal facility (South Kensington campus) or the Aston University central animal facility under specific pathogen-free conditions. The mice were provided with food and water and exposed to a 12-h light–dark cycle. All mice were handled in compliance with UK Home Office and Imperial College regulations on animal care and welfare (Animals (Scientific Procedures) Act 1986). Allergic airway disease was induced using a previously described protocol [3]. In brief, on 5 days per week over the course of five consecutive weeks, mice (n = 15) were anesthetized with isoflurane (Sigma-Aldrich) before being challenged with house dust mite allergen (HDM). HDM extract (Greer Laboratories, USA or Citeq, The Netherlands) was suspended in sterile phosphate buffered saline (PBS) at a final concentration of 2.5 mg/ml. Ten µl of the solution was administered intranasally; control mice (n = 15) received 10 µl of sterile PBS using the same protocol. LIT-927 was obtained from Axon Medchem (Groningen, The Netherlands) and was diluted to 197 ng/ml in methyl-β-cyclodextrin (Sigma) 10% w/v and delivered intranasally (10 µl) immediately before allergen exposure. Immediately after PBS/HDM administration, respiratory distress symptom scores were collected by assessing for the presence of sneezing, nose rubbing, labored breathing, reduced locomotion, and audible wheezing (one point each to a maximum score of 5).

### Sample collection

All mice were euthanised humanely using a pentobarbital overdose administered intraperitoneally. Different samples were then collected from individual mice for separate analyses. Bronchoalveolar lavage fluid was harvested for immune cell counts. Procedures requiring materials from mice included lung section immunostaining, flow cytometry and cell culture. The lungs were surgically removed and processed for hematoxylin and eosin or immunostaining as described below. Whole lungs were taken and digested for analysis via flow cytometry or immunomagnetic isolation of CD146+ cells for use in cell culture.

### Preparation of single cell suspension from mouse lung

Bilateral thoracotomy was performed to expose the pleural cavity. A small incision was made in the left ventricle, and 10 ml ice cold PBS was flushed through the circulatory system via the right ventricle using a 21G needle and 10 ml syringe. The lungs were removed and placed in an Eppendorf tube with 0.5 ml DMEM pen/strep (1%). Lungs were finely minced with scissors, and 0.5 ml DMEM pen/strep with 0.195WU/ml Liberase™ thermolysin (Roche Diagnostics) and DNAse I (Sigma-Aldrich) added before incubating at 37 °C for 45 min.

Reaction was terminated using 1 ml FBS with EDTA (5 mM), and samples were kept on ice hereafter. Digested tissue was mechanically dissociated by passing through a 100 µm cell strainer (Miltenyi) using a syringe plunger, washed twice in RPMI buffer (RPMI, pen/strep, HEPES (25 mM), EDTA (5 mM) and FBS (10%), 1200 rpm, 4 °C, 6 min) before filtering through a 70 µm filter (Miltenyi) to create a single cell suspension.

### Staining of lung cells for flow cytometry

Cells were diluted to 10–50 × 10^6^ cells/ml in staining buffer (PBS, 10% FBS, EDTA (5 mM), and F_c_ receptor blocked with anti-CD26/32 (1:100, Biolegend) for 10 min on ice. Cells were aliquoted into 100 µl/well V-bottomed 96-well plates (Corning), centrifuged (1200 rpm, 5 min, 4 °C), and resuspended in staining buffer containing antibodies pre-conjugated to fluorophores (see Table 1) for 30 min ice in the dark. Cells were washed twice to remove unbound antibody before fixing in 100 µl/well IC fixation buffer (eBioscience) for 20 min ice in the dark, washing, and re-suspending in 200 µl PBS. Stained samples were stored at 4 °C, in the dark.

**Table 1:** Pericyte panel for flow cytometry

Antibody	Target	Clone	Fluorophore	Dilution (μl/100 μl)	Supplier
CD31	Endothelial cells	390	FITC*	2	Biolegend
			PerCp-Cy5.5**	1.25	Biolegend
CD45	Pan-leukocytes	30-F11	PerCp-Cy5.5	1.25	Biolegend
CD146	Pericytes	ME-9F1	PE-Cy7	1.25	Biolegend
			FITC	1	
PDGFRβ	Pericytes	APB5	APC	5	Biolegend
Ter119	Erythrocytes	TER-119	PerCP-Cy5.5	1.25	Biolegend

^*^Used as part of dump channel with the LEGENDScreen™

^**^Used for endothelial cell exclusion in all other experiments

### Flow cytometry

Single color compensation controls using VersaComp antibody capture beads (Beckman Coulter) and Fluorescence Minus One (FMO) controls were prepared at time of staining following the same protocol. Stained controls were stored at 4 °C in the dark. All flow cytometry analyses were performed on a BD LSR Fortessa or Cytoflex flow cytometer equipped with 405 nm, 488 nm (530/30—FITC/AF488, 695/40—PerCp-Cy5.5), 561 nm (585/15—PE, 780/60—PE-Cy7) and 640 nm (670/14—APC) lasers and filters. Data were analyzed using FlowJo (Treestar) software. Detail on the antibodies used are provided in Tables 1 and 2.

**Table 2:** Experimental markers to assess pulmonary pericyte phenotype by flow cytometry

Antibody	Clone	Fluorophore	Dilution (μl/100 μl)	Supplier
CD13	R3-242	PE	2	BD Pharmingen
CXCR4	L276F12	PE	2.5	Biolegend
Podoplanin	8.1.1	PE	2	Biolegend

### Pericyte enrichment

For LEGENDScreen™ and cell culture studies, murine lung was enriched for MSC by depleting CD45^+^, ter119^+^ cells using the EasySep™ Mouse Mesenchymal Stem/Progenitor Cell Enrichment Kit with the EasySep™ magnet.

### LEGENDScreen™ antibody panel

Enriched cells were blocked, stained with viability dye (as described) and resuspended at 10 × 10^6^ cells/ml in Biolegend staining buffer with the pericyte panel (see Table 1). Lyophilized PE-conjugated antibody cakes were dissolved in 25 µl Biolegend staining buffer. Then, 75 µl of the cell suspension was added per well containing antibody (LEGENDScreen™, Biolegend) and incubated for 30 min on ice in the dark. Cells were fixed and plates stored as described above.

### Pericyte culture and immunohistochemistry

MSC enriched lung suspensions were plated onto one T25 culture flask/mouse in pericyte growth medium (PromoCell GmbH). After 1 week, cultures were washed with warm, sterile PBS and pericyte medium replaced. Medium was changed twice per week until 80–90% confluent, at which point cells were trypsinized (0.25% Trypsin, EDTA, Sigma Aldrich) for 5 min at 37 °C. Trypsin was neutralized with DMEM 10% FBS, 1% pen/strep, and cells plated onto a T75 flask. Following three passages, cells were plated onto gelatin-coated coverslips at 0.1 × 10^6^ cells/well in 12-well plates and cultured in pericyte growth medium until 90% confluent.

For immunohistochemistry, cells were washed thrice (5 min) in PBS before fixation in 3% PFA for 15 min RT. Cells were washed, then non-specific binding was blocked using 5% normal goat serum in 0.3% Triton-X-100/PBS for 1 h. Cells were incubated with primary antibodies (Table 3) for 2 h, then washed and incubated with the appropriate secondary antibodies for 1 h RT. Finally, cells were washed and coverslips were mounted on microscope slides using Fluoroshield (Sigma-Aldrich) containing 4',6-diamidino-2-phenylindole (DAPI) to provide nuclear staining.

**Table 3:** Antibodies used in immunostaining

Antibody	Host species	Clone	Dilution	Supplier
PDGFRβ	Rat	APB5	1:500	Abcam
NG2	Rabbit	AB5320	1:250	Millipore
CD31	Hamster	2H8	1:500	Abcam
CXCR4	Rat	2B11	1:200	eBioscience
α-SMA	Mouse	1A4	1:1000	Sigma-Aldrich

### Transwell migration assay

The migration of pericytes toward CXCL12 (Peprotech EC) was assessed using Transwell assays; PDGF-B (300 ng/ml; Peprotech EC) was used as the positive control, while DMEM with nothing added served as the negative control. In some experiments, CXCL12 binding to CXCR4 was blocked with LIT-927 (Axon Medchem) or the downstream signaling pathway of CXCR4 was inhibited using the p38 MAPK inhibitor SB203580 (Sigma-Aldrich). The specific mediators were diluted to the desired concentration using serum-free DMEM (Sigma-Aldrich). Three hundred µl of each ligand was then added to a designated well of a 12-well plate. Pericytes from culture were diluted to a concentration of 5 × 10^5^ cells/ml. One hundred µl of cells were then added to the Transwell inserts (Corning), which were placed in each individual well. The plates were incubated at 37 °C and in a 5% CO_2_ atmosphere for 24 h. After 24 h, medium containing residual cells was removed from the upper chamber of the Transwell using a pipette, then a cotton swab, before fixation in methanol for a minimum of 10 min. Filters were removed from each insert, washed in ddH_2_O and mounted on individual slides using Fluoroshield containing DAPI to provide nuclear staining. Slides were stored at − 20 °C until image acquisition. Cells were visualized at 358 nm using a Leica DM2500 fluorescence microscope (Leica Microsystems, Milton Keynes, UK). Cells on the inferior side of the filter were imaged at 70× magnification by acquiring Z stack images comprised of 4 images each separated by 2 µm. Images were then processed using ImageJ software (NIH). Separate ‘particles’ were counted as single cells.

### Lung histology

Lungs were inflated with 10% formalin, kept in 10% formalin overnight, then transferred to 100% ethanol. Tissues were paraffin embedded and cut at a thickness of 3 µm. Sections were then stained with hematoxylin and eosin using a standard protocol (Sigma-Aldrich).

### Immunostaining of lung sections and tracheobronchial whole mounts

Whole lungs taken from mice were stored in sucrose solution to cryopreserve the sample. Sucrose was rinsed off using PBS. Whole lungs were then embedded in TissueTek OCT (Sakura) and frozen at − 80 °C. Ten micrometer-thick sections were then cut using a cryostat (Leica) and mounted on Superfrost Plus slides (Fisher Scientific). The slides were stored at -80 °C. Prior to staining, slides were warmed to room temperature. A hydrophobic marker was used to outline the tissue sections. Sections were incubated for a minimum of 2 h in 5% NGS and 0.3% Triton-X diluted in PBS to prevent non-specific binding and to permeabilize the section. Following blocking, slides were washed in PBS and incubated overnight with the designated primary antibodies (Table 3). Slides were once more washed in PBS before incubation with the appropriate secondary antibodies specific to the relative species of each of the three primary antibodies. Additionally, for polyclonal antibodies, negative reagent controls were carried out by staining sections without the primary antibodies. Sections were incubated in secondary antibodies for a minimum of 2 h (Table 3). Specimens were then washed in PBS before mounting in Fluoroshield containing DAPI.

For whole-mount immunostaining of mouse tracheas and bronchi, the large airways were dissected from the larynx to the lungs, cleaned of extraneous connective tissue and mounted on Kwikgard™-coated (WPI, Sarasota, Fl, USA) 6-well culture plates. Non-specific binding was blocked by incubating trachea sections in 5% NGS/0.3% Triton-X 100 in PBS O/N. Tracheas were cut in half lengthways before the left and right tracheobronchial sections were separated and stained for pericytes and endothelial cells using the appropriate primary and secondary antibodies O/N at RT (Table 3). Sections were washed thrice in PBS and mounted using Fluoroshield. The antibodies were titrated to determine the optimal concentrations.

Haematoxylin and eosin stained lung sections were visualized and images using an EVOS brightfield microscope (ThermoFisher). Immunostained lung sections were visualized and images were captured using a TCS SP8 FALCON Leica confocal microscope equipped with a DMOD 405 laser and white light laser with tunable excitation from 470 to 670 nm. Slides were imaged in sequential mode using a HC PL APO 40X /0.95 dry objective. The following settings were used: DAPI—excitation 405 nm laser line, emission detected between 410 and 609 nm; Alexa 488—excitation 499 nm laser line, emission detected between 504 and 568 nm; Alexa 546—excitation 557 nm laser line, emission 562–776 nm; Alexa 647—excitation 635 nm laser line, emission 658–775 nm. Tracheobronchial whole mounts were visualized and images were captured using a Zeiss LSM0-510 inverted confocal microscope equipped with Diode 450 nm, Argon 488 nm, HeNe 545 nm and HeNe 633 nm lasers and EC Plan-Neofluar 10 × 0.30 M27 and EC Plan-Neofluar 20 × 0.50 M27 objectives with an X-Cite Series 120 halogen lamp fluorescent light source. Images were processed using LAS X software (Leica).

### Molecular modeling

A model of the full-length CXC4R receptor was generated by MODELLER [20] using the PDB structures 3OE0 and 2N55 as templates. MODELLER generated one thousand initial models, which were refined and rescored using ROSETTA using the implicit membrane forcefield [21]. The best scoring ROSETTA model was used in subsequent docking studies. LIT-927 was docked into the average NMR structure taken from PDB:4UAI using AUTODOCK [22] under default settings. Default ligand and receptor-based charges, as defined in AUTODOCKTOOLS [22], were used for the 100 independent docking runs. The results were clustered into 2 Å bins. The largest cluster with the lowest docking score for CXCL12:LIT-297 complex was retained for further docking studies with the CXCR4. The entire surface of the CXCL12 was used in the docking run. GRAMM [23] in low-resolution mode was used to dock CXCL12 alone or the CXCL12:LIT-27 pre-docked complex into the full-length model of the CXCR4. Five thousand docking runs were completed for each ligand, with the resulting complexes being refined using ROSETTA. The lowest 50 structures from each docking run were clustered using a 2 Å cut-off. The mid structure from the largest cluster from each docking run was visually inspected to determine the role of LIT-927 in the binding of CXCL12 to CXCR4.

### Statistical analysis

Data were analyzed with GraphPad Prism Software version 8 (GraphPad Software, San Diego, CA). Sample size calculations were performed before in vivo experiments to ensure adequately powered results, with a minimal n = 5 for morphometric assessments and n = 7 for flow cytometry. Statistical analysis was performed by testing for normality using the Shapiro–Wilk test, followed by Student’s t-test or one-way ANOVA with a subsequent Tukey’s post hoc test where appropriate. Differences were considered statistically significant when P < 0.05. Results are presented as mean ± SEM.

## Results

### Marker expression on murine pulmonary pericytes

**Fig. 1:**
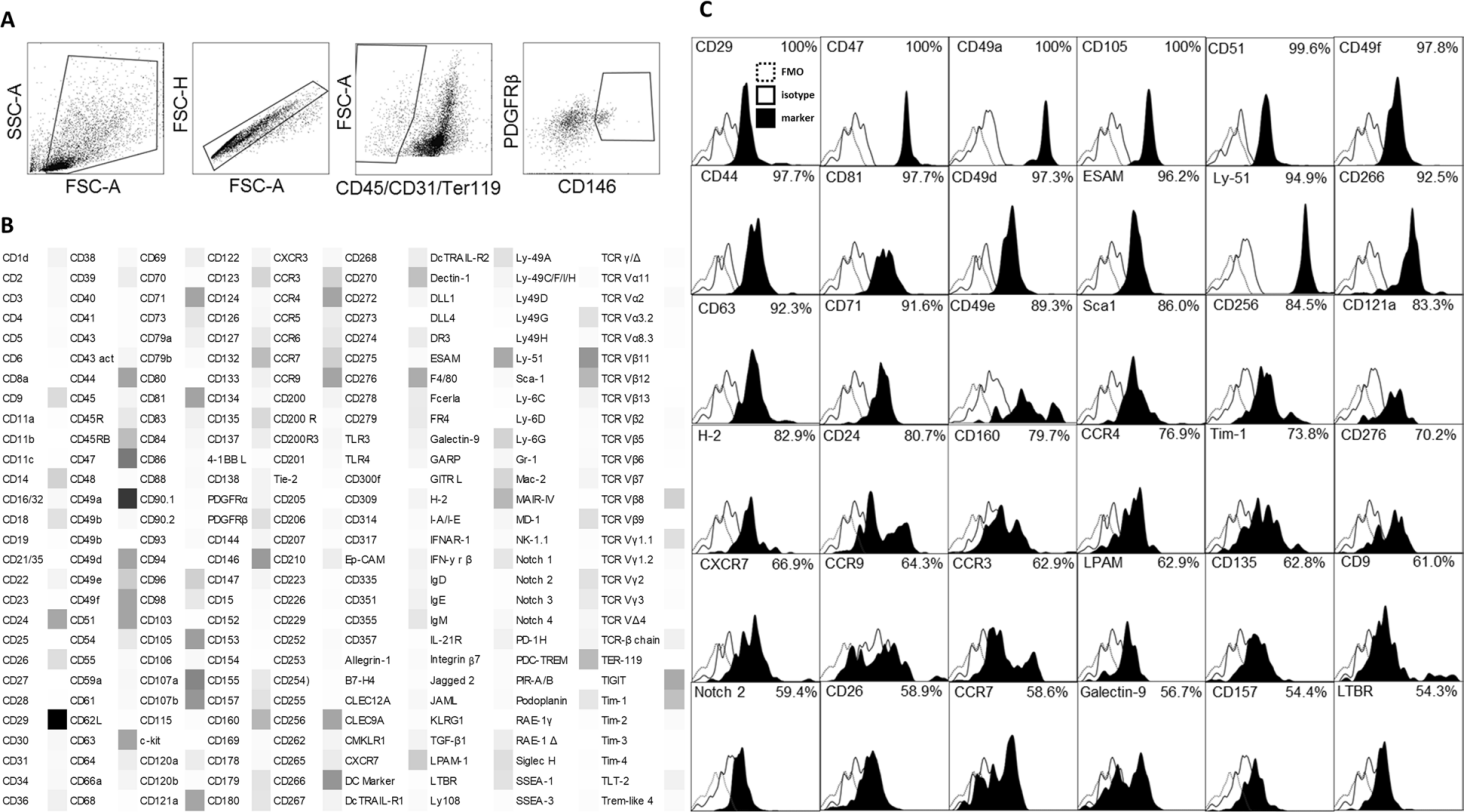
Pulmonary pericyte characterization by flow cytometry. The lungs of female C57/Bl6 mice (6–8 weeks old) were removed and stained by flow cytometry. **A** Pulmonary pericytes were identified as CD45-/CD31-/Ter119-/PDGFRβ+/CD146+ cells. **B** Further analysis using the Biolegend LegendScreen™ indicated the absence of hematopoietic cell markers and the presence of mesenchymal cell markers on these cells. **C** Histograms indicate the distribution of expression of highly expressed markers. FMO: fluorescence minus one control; FSC: forward scatter; SSC: side scatter

As pericytes are considered an MSC-like cell residing in the perivascular niche of tissue throughout the body, including the lung, the expression of classic MSC markers were compared with antigens that pericytes expressed in this assay. For characterization of pericyte cell surface markers, murine pulmonary pericytes determined as being CD45^−^/CD31^−^/ter119^−^, CD146^+^/PDGFRβ^+^ were screened for 252 markers using the LEGENDScreen™ panel (Fig. 1A). This assay revealed that murine pulmonary pericytes highly express the integrins CD29 and CD49a, both of which have been reported to be expressed by MSC [24]. Other highly expressed markers were CD47, CD105, CD266 and Ly-51. Pericytes were also found to express CD107a and CD107b, which are lysosomal degranulation markers also known as LAMP1 and LAMP2 (Fig. 1B, C). This screen was used to define our gating strategy for pulmonary pericytes in subsequent experiments.

### Pericyte marker expression is not modulated by chronic allergic inflammation

**Fig. 2:**
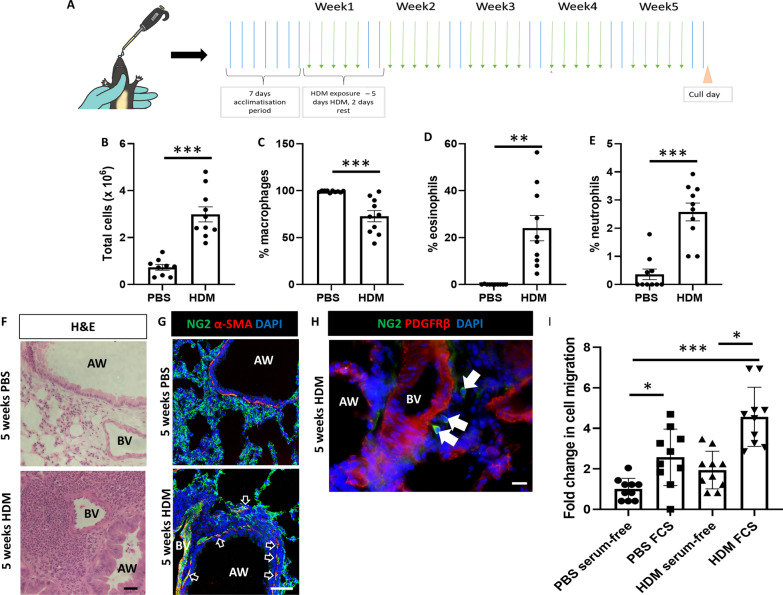
Pericytes accumulate in the airway wall in response to chronic allergic inflammation. **A** Female C57/Bl6 mice (6–8 weeks old) were subjected to intranasal delivery of sterile PBS (10 µl) or house dust mite extract (HDM; 25 µg in 10 µl) 5 days a week for 5 consecutive weeks. At the end of the protocol, the lungs were removed and bronchoalveolar lavage (BAL) fluid was collected. **B** Total inflammatory cell infiltrates were enumerated and **C**–**E** immune cell differentials were determined using hematoxylin and eosin stained cytospin preparations of BAL fluid. **F** Hematoxylin and eosin staining of paraffin-embedded lung sections demonstrated perivascular and peribronchial inflammatory infiltration in HDM exposed mice. **G** Immunostaining of frozen lung sections revealed peribronchial accumulation of NG2+ pericytes (green) coexpressing αsmooth muscle actin (αSMA, red) in the airway wall of HDM-exposed mice (black arrows). **H** Immunostaining for the pericyte markers NG2 (green) and PDGFRβ (red) revealed pericyte uncoupling from the pulmonary vasculature of HDM-exposed mice (white arrows). **I** Lung tissue was digested and pericytes were obtained by magnetic sorting, then subjected to a Transwell assay using fetal calf serum (FCS) as the chemoattractant. n = 10 per group, representative of four independent experiments. *p < 0.05, **p < 0.01, ***p < 0.001. Scale bar 25 µm. AW: airway; BV: blood vessel; HDM: house dust mite; PBS: phosphate-buffered saline

A commercially available extract of the house dust mite *Dermatophagoides pteronyssinus* (HDM) was delivered intranasally 5 days/week for 5 consecutive weeks in order to elicit an immune response comparable to human allergic asthma (Fig. 2A). HDM exposure increased the number and variety of immune cells present in the bronchoalveolar fluid samples (Fig. 2B), particularly increasing the percentage of eosinophils and neutrophils, with an accompanying decrease in the relative proportion of macrophages (p < 0.01; Fig. 2C–E). Hematoxylin and eosin staining of lung tissue revealed robust perivascular and peribronchial accumulation of inflammatory cells in HDM-exposed mice (Fig. 2F); additionally, immunostaining of lung sections demonstrated the accumulation of NG2 + pericytes in the airway wall of HDM-exposed mice (Fig. 2G) and evidence of pericyte uncoupling from NG2/PDGFRβ blood vessels adjacent to large airways (Fig. 2H). In parallel studies, lung tissue was digested and pericytes were obtained by magnetic sorting, then subjected to a Transwell assay using fetal calf serum (FCS) as the chemoattractant. Pericytes isolated from HDM-exposed mice migrated more readily than healthy pericytes both towards the FCS stimulus, as well as toward medium without a chemoattractant (p < 0.001; Fig. 2I).

### Pericytes acquire a migratory phenotype following HDM exposure in vivo

**Fig. 3:**
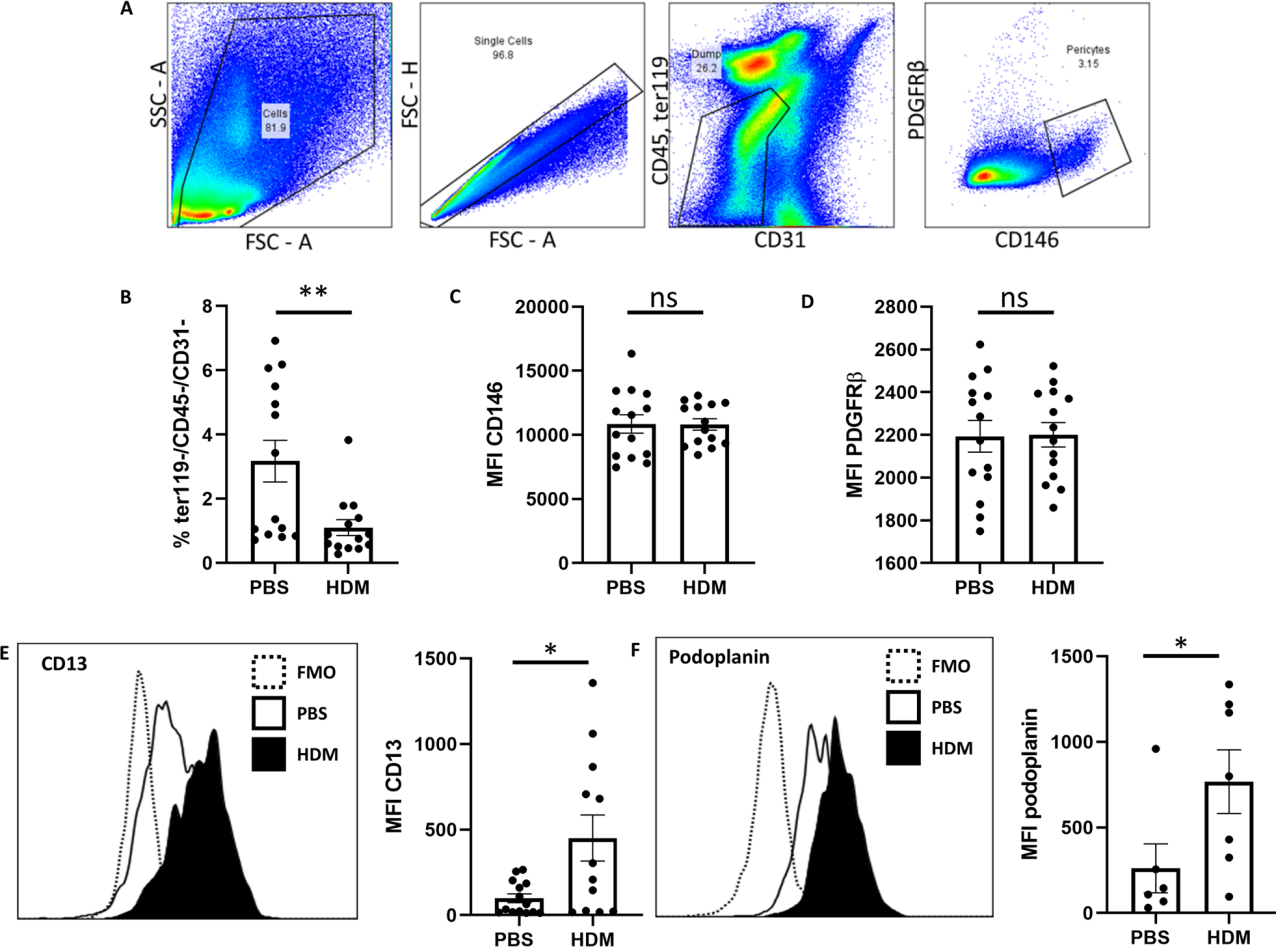
Pericytes acquire a migratory phenotype following HDM exposure in vivo. Female C57/Bl6 mice (6–8 weeks old) were subjected to intranasal delivery of sterile PBS (10 µl) or house dust mite extract (HDM; 25 µg in 10 µl) 5 days a week for 5 consecutive weeks. **A** Lungs were processed into a single cell suspension and submitted to flow cytometric analysis. Pericytes were defined as whole, single cells (FSC, SSC), negative for the markers ter119, CD31, and CD45, and positive for the markers CD146 and PDGFRβ. **B** The proportion of ter119-/CD31-CD45- cells and the median fluorescence intensity of CD146 (**C**) and PDGFRβ (**D**) were determined using FlowJo software. n = 14 per group, representative of three independent experiments. **E**, **F** Pericytes were also assessed by flow cytometry to determine the median fluorescence intensity of CD13 and podoplanin on pulmonary pericytes using FlowJo software. n = 14 per group, representative of three independent experiments. * p < 0.05, ** p < 0.01, *** p < 0.001. FCS: fetal calf serum; FMO: fluorescence minus one control; FSC: forward scatter; HDM: house dust mite; MFI: median fluorescence intensity; PBS: phosphate-buffered saline; SSC: side scatter

To determine the mechanism responsible for the enhanced migratory capacity of pulmonary pericytes obtained from the lungs of HDM-exposed mice, pericytes (negative for CD31, CD45, and Ter119) were subsequently assessed by flow cytometry (Fig. 3A) for key surface markers defining the pericyte phenotype (CD146, PDGFRβ) and markers associated with cell migration (CD13, podoplanin; Fig. 3E, F). Pericytes represented a smaller proportion of lung cells in HDM-exposed lungs (p < 0.01; Fig. 3B) due to the influx of inflammatory cells. However, the expression of the pericyte surface markers PDGFRβ and CD146 were unchanged following the induction of allergic airway disease (Fig. 3C, D). To further explore the induction of a migratory phenotype in pulmonary pericytes, the expression of the cell migration markers CD13 (Fig. 3E) and podoplanin (Fig. 3F) were assessed by flow cytometry. Pericytes in the lungs of HDM-exposed mice expressed significantly higher levels of these markers of migratory cells, assessed by median fluorescence intensity (MFI; p < 0.05).

### Pericyte migration is mediated via the CXCL12/CXCR4 pathway

**Fig. 4:**
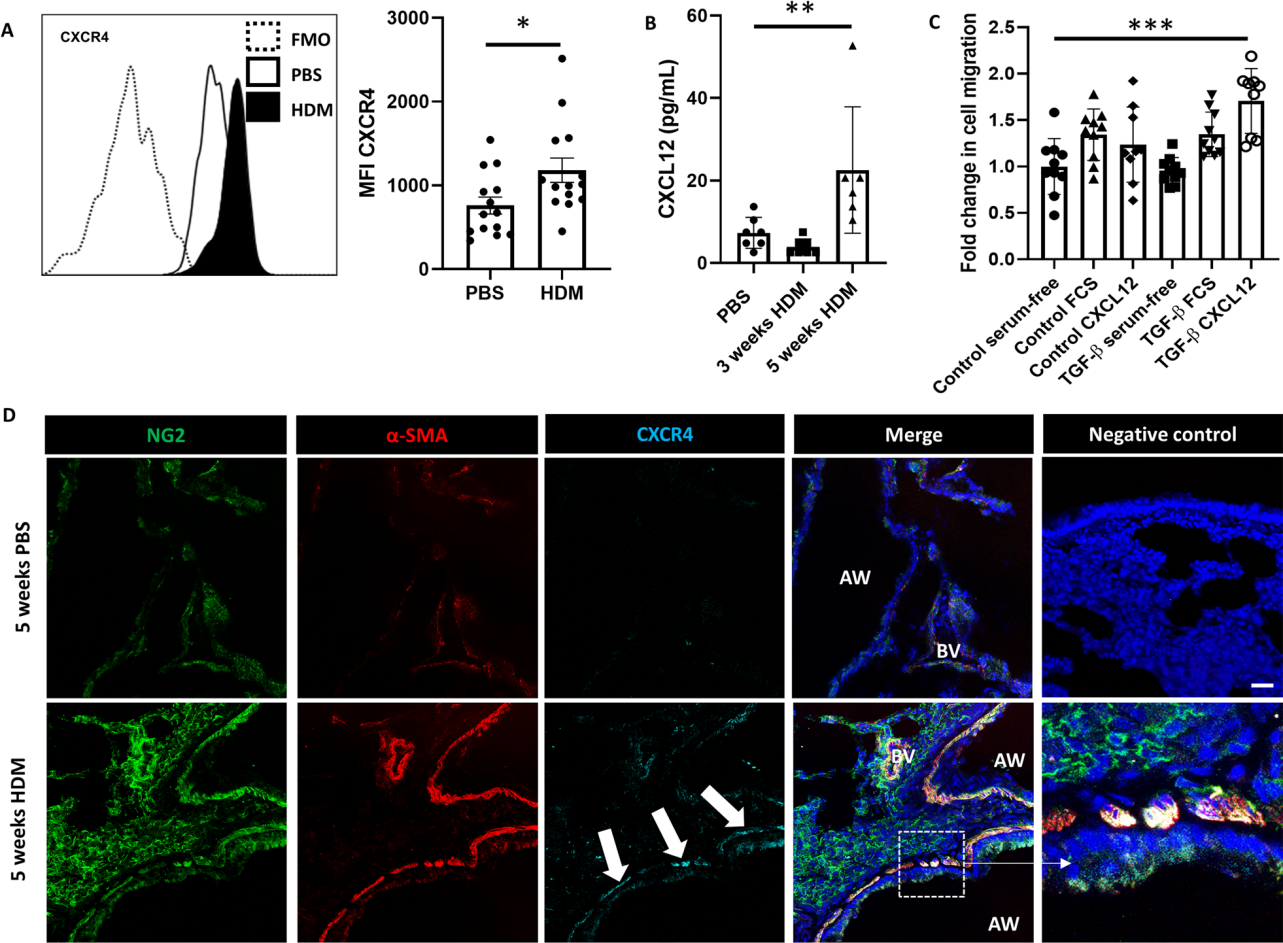
Pericyte migration is mediated via the CXCL12/CXCR4 pathway. Female C57/Bl6 mice (6–8 weeks old) were subjected to intranasal delivery of sterile PBS (10 µl) or house dust mite extract (HDM; 25 µg in 10 µl) 5 days a week for 5 consecutive weeks. **A** At the end of the protocol, the lungs were removed and pericytes were assessed by flow cytomtry to determine the median fluorescence intensity of CXCR4 using FlowJo software. n = 14 representative of three independent experiments; * p < 0.05. **B**) Bronchoalveolar fluid from PBS control mice and mice exposed to HDM for either 3 weeks or 5 weeks was submitted to ELISA to assess levels of CXCL12. n = 7–8 per group from two independent experiments; **p < 0.01. **C** Healthy human pericytes were treated with TGF-β in vitro (10 ng/ml for 7 days) and subjected to Transwell assays using fetal calf serum (FCS) or CXCL12 (300 ng/well) as the chemoattractant. n = 8–10 per group, representative of three independent experiments; ***p < 0.001. **D** Lung sections obtained from PBS control and HDM-exposed mice were stained for the pericyte marker NG2 (green), the mesenchymal cell marker α-smooth muscle actin (α-SMA; red) and CXCR4 (cyan) to assess the infiltration of CXCR4+ pericytes into airway smooth muscle bundles (white arrows). Scale bar 25 µm. AW: airway; BV: blood vessel; HDM: house dust mite; LIT: LIT-927 (CXCL12 neutraligand); PBS: phosphate-buffered saline; VEH: vehicle control

Pericytes were isolated from murine whole lung digests using the gating strategy described above. Pericyte CXCR4 expression was significantly increased in the lungs of mice exposed to HDM (p < 0.05; Fig. 4A), as were levels of its cognate ligand CXCL12 (p < 0.01; Fig. 4B), detected in bronchoalveolar fluid. To further examine the mechanisms of increased pericyte migration to CXCL12, Transwell assays were performed on human placental pericytes (HPLPC) treated with TGF-β. As shown in Fig. 4C, pericytes treated with TFG-β migrated more readily to CXCL12 compared to untreated pericytes (p < 0.001). CXCR4 expression was also assessed in situ by performing immunofluorescent staining on lung sections from PBS- and HDM-exposed mice (Fig. 3C). Immunostaining of mouse lung sections for CXCR4, the pericyte marker NG2, and the smooth muscle/myofibroblast marker α-smooth muscle actin (α-SMA) indicated limited expression of CXCR4 in control mice. In contrast, there was a clear increase in the accumulation of CXCR4 expressing cells in the airway wall of HDM-exposed mice; CXCR4 expression colocalised with NG2+ cells adjacent to airway smooth muscle bundles (Fig. 4D).

### Topical treatment with LIT-927 has no effect on HDM-induced airway inflammation but decreases respiratory symptom scores

**Fig. 5:**
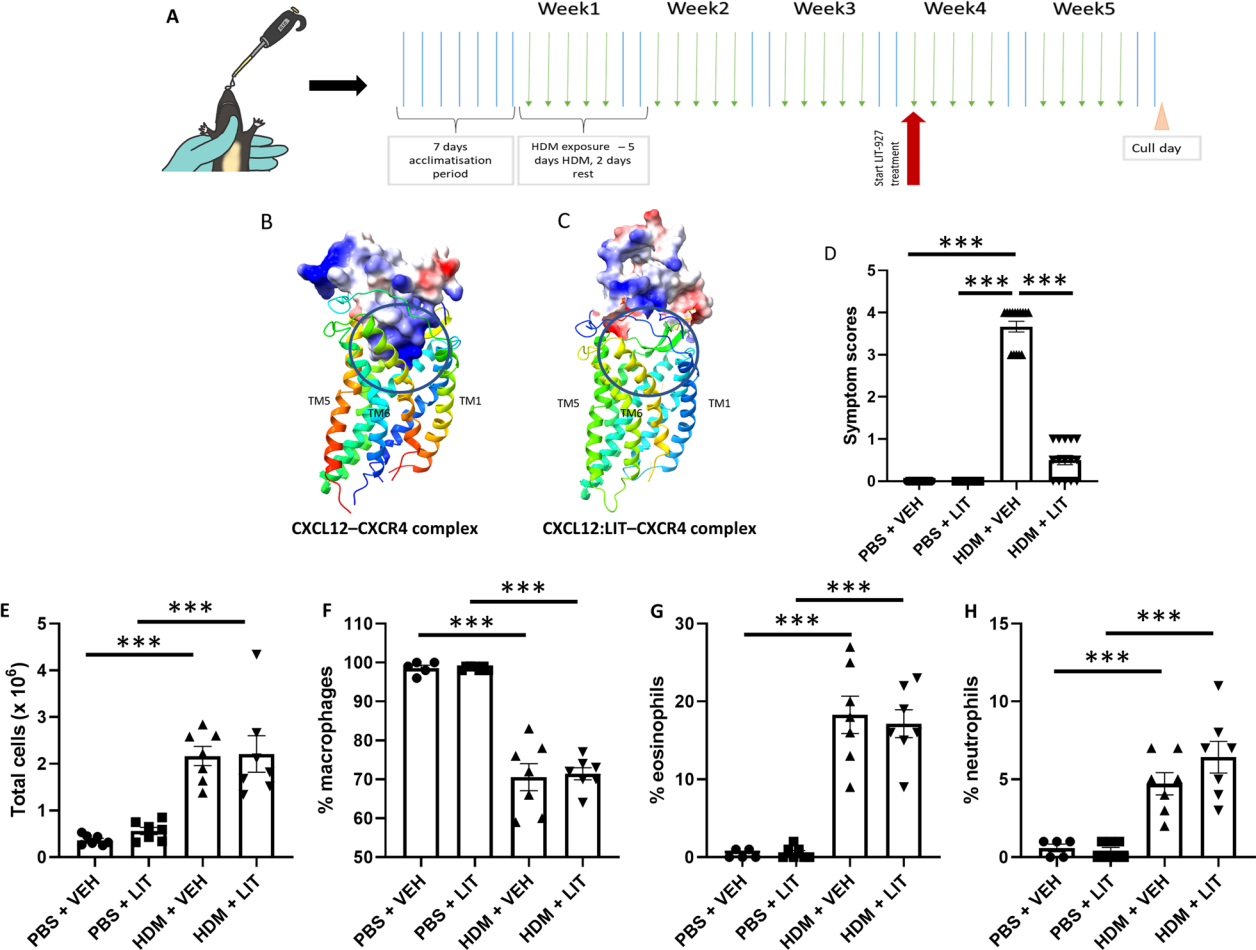
Topical treatment with LIT-927 has no effect on HDM-induced airway inflammation but decreases respiratory symptom scores. Female C57/Bl6 mice (6–8 weeks old) were subjected to intranasal delivery of sterile PBS (10 µl) or house dust mite extract (HDM; 25 µg in 10 µl) 5 days a week for 5 consecutive weeks. At the end of the protocol, the lungs were removed and bronchoalveolar lavage (BAL) fluid was collected. **A** Schematic diagram of the schedule of allergen and drug delivery. **B**, **C** The orientation of the CXCL12 alone or CXCL12:LIT927 complex and the CXCR4. The receptor is depicted in ribbon format and colored N > C using rainbow coloring. The ligand is represented as a surface map and colored according to charge distribution. The circle highlights ligand penetration into the receptor core in either the undocked (CXCL12 only) or pre-docked (CXCL12:LIT-927) (**B**) the orientation of CXCL12 complex with CXCR4 (**C**) the orientation of the pre-docked CXCL12:LIT-927 complex with CXCR4. **D** Symptom scores were monitored throughout the 5-week protocol. Total inflammatory cell infiltrates were enumerated (**E**) and immune cell differentials (**F**–**H**) were determined using hematoxylin and eosin stained cytospin preparations of BAL fluid. n = 15 per group from two independent experiments; ***p < 0.001. HDM: house dust mite; LIT: LIT-927 (CXCL12 neutraligand); PBS: phosphate-buffered saline; VEH: vehicle control

In order to investigate the role of the CXCL12-CXCR4 signaling axis in pericyte accumulation in the airway wall, mice were subjected to the HDM allergic airway disease model and treated with LIT-927, a neutraligand of the chemokine CXCL12, for the final 2 weeks of the five-week HDM exposure protocol (Fig. 5A). HDM extract was delivered intranasally 5 days/week for a period of 5 weeks. After three weeks of HDM exposure (before the peak in CXCL12 expression in the lung; Fig. 4B), CXCL12 neutralization was initiated with LIT-927, delivered intranasally to isoflurane-anesthetized mice immediately prior to HDM delivery (LIT groups). Vehicle control mice (VEH groups) received PBS containing 0.05% cyclodextrin as the drug carrier. LIT-927 treatment was carried out for 2 weeks concomitantly with allergen exposure. To investigate how LIT-927 influences the binding of CXCL12 to the CXCR4, an incremental docking approach was used (Fig. 5B, C). Ligand docking poses between CXCL12 only and the CXCL12:LIT-927 pre-docked complex were generated by GRAMM and subsequently refined and scored using ROSETTA. Visual inspection of the CXCL12–CXCR4 complex highlighted critical interactions between the N-terminus of the receptor and the chemokine (Fig. 5B). Strong hydrogen bonds were generated between Tyr^21^ (CXCR4 N-terminus) and a cluster of Asn residues in CXCL12 (Asn^22^, Asn^44^, and Asn^45^). Furthermore, salt bridges between CXCR4 Asp^6.58^: CXCL12 Arg^8^ and CXCR4 GLU^7.28^: CXCL12 Arg^12^ were also observed to accompany a strong electrostatic and steric set of interactions between the remaining portions of the receptor and ligand. However, the pre-docking of CXCL12 with LIT-927 before docking to CXCL4 resulted in a drastically altered conformation. The key salt bridges between the chemokine and the receptor were not present, and the CXCL12:LIT-927 complex did not penetrate the core of the receptor (Fig. 5C). Upon further inspection, LIT-927 was bound to a cavity on the surface of CXCL12 enclosed by Asn^22^, Asn^44^, and Asn^45^, preventing fundamental interactions such as with Tyr21, as observed in the docked complex in the absence of LIT-927.

Symptoms of airway inflammation and respiratory distress in mice were monitored daily following PBS or HDM exposure, with observations including sneezing, nose rubbing, hunched posture, labored breathing, and audible wheezing (one point each for a maximum of five points on the scale) (Fig. 5D). No signs of respiratory distress were observed in PBS-exposed control mice given either LIT-927 or the vehicle control (average score of 0 in both groups). Conversely, mice exposed to HDM extract began to display signs of respiratory inflammation such as sneezing and nose rubbing by 3 weeks of exposure (average score of 1.8); these symptoms worsened over the course of the experiments until achieving an average of 3.7 points by 5 weeks of HDM exposure. However, in the group of mice were treated with LIT-927 starting at week 3, respiratory distress scores began to decrease within 1 week of initiating LIT-927 treatment (average score of 1.5 vs. 2.8 in HDM-exposed mice given the vehicle). Respiratory distress scores decreased nearly to baseline 2 weeks after the first dose of LIT-927 (average score of 0.5 vs. 3.7 in HDM-exposed vehicle-treated mice; p < 0.001; Fig. 5D). Following the humane cull of the mice after 5 weeks of PBS/HDM exposure + VEH/LIT treatment, bronchoalveolar lavage was collected, and cell counts and differentials were performed (Fig. 5E–H). Mice exposed to HDM, regardless of LIT-927 or drug vehicle treatment, had a significantly higher number of immune cells in the BAL (p < 0.001; Fig. 5E), with a significantly lower proportion of macrophages (p < 0.001; Fig. 5E). Cell differentials indicated the influx of granulocytes, primarily eosinophils (p < 0.001; Fig. 5G) with smaller numbers of neutrophils (p < 0.001; Fig. 5H), into the airways of HDM-exposed mice. LIT-927 treatment had no impact on immune cell differentials.

### LIT-927 impedes pericyte uncoupling from the airway microvasculature

**Fig. 6:**
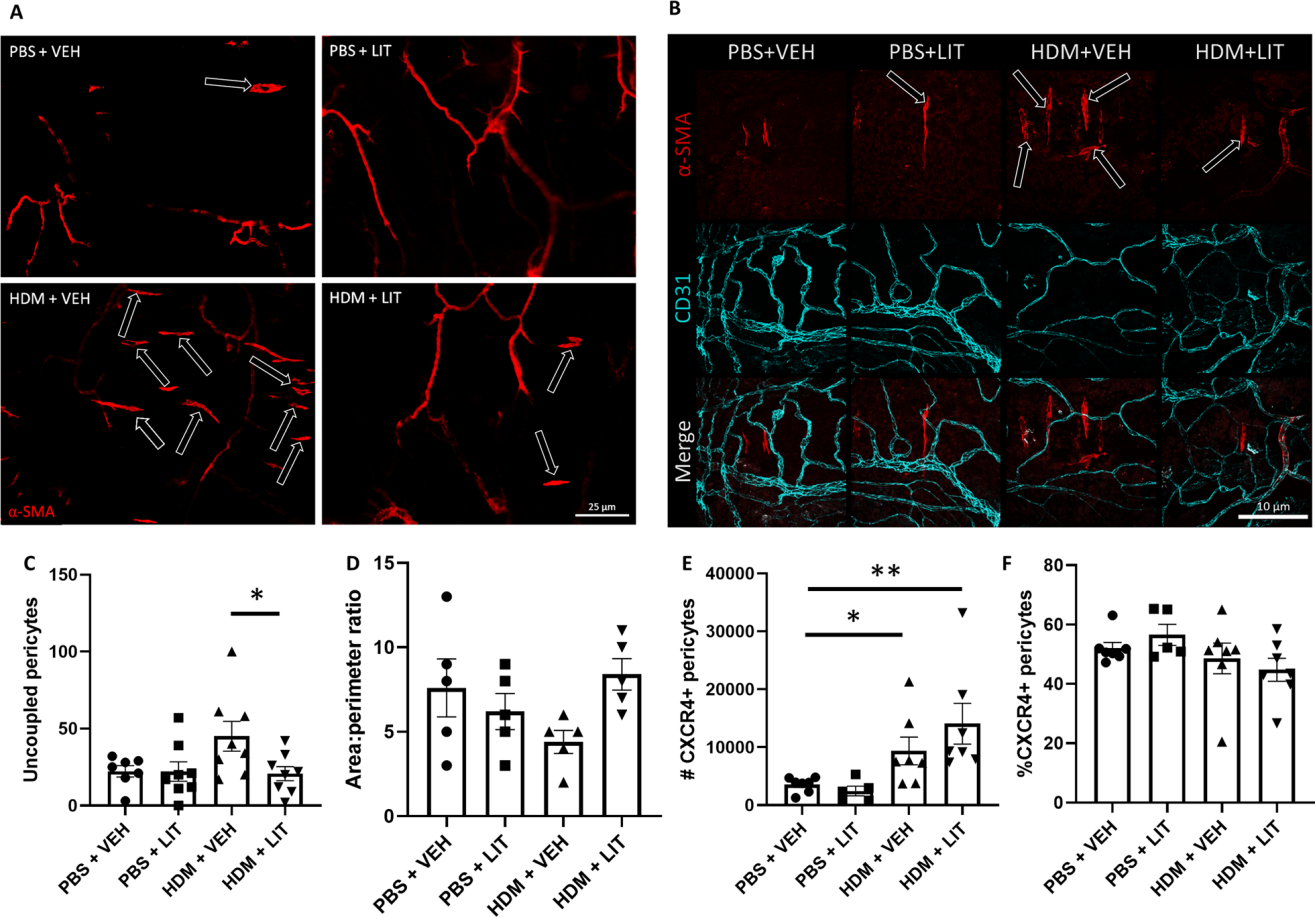
LIT-927 impedes pericyte uncoupling from the airway microvasculature. Female C57/Bl6 mice (6–8 weeks old) were subjected to intranasal delivery of sterile PBS (10 µl) or house dust mite extract (HDM; 25 µg in 10 µl) 5 days a week for 5 consecutive weeks. **A**, **B** At the end of the protocol, the trachea and bronchi were collected, cleaned, and stained as a whole mount to perform a three-dimensional analysis of uncoupled pericytes mobilized, uncoupled pericytes (pericytes that have dissociated from the microvasculature). Tracheobronchial whole mounts were stained for the mesenchymal cell marker α-smooth muscle actin (α-SMA; red) and the endothelial cell marker CD31 (cyan). Scale bar 25 µm (**A**) and 10 and µm (**B**). **C** Uncoupled α-SMA+ cells (example indicated by black arrows) were counted and **D** the area:perimeter ratio was calculated. n = 5–8 per group from two independent experiments; *p < 0.05. **E** Lungs were processed into a single cell suspension and submitted to flow cytometric analysis to determine the number and proportion of CXCR4 + pericytes. n = 7 per group, representative of two independent experiments; *p < 0.05, **p < 0.01. HDM: house dust mite; LIT: LIT-927 (CXCL12 neutraligand); PBS: phosphate-buffered saline; VEH: vehicle control

The large airways (trachea and bronchi) were obtained from mice exposed to PBS/HDM for 5 weeks and treated with LIT-927/VEH for the last 2 weeks of the experiment and stained as a whole mount with α-SMA to indicate migratory, uncoupled pericytes (pericytes that have dissociated from the microvasculature) and CD31 to label blood vessels. Figure 6A, B show representative images pericytes associated with the pulmonary vasculature, with considerably higher numbers of α-SMA+ pericytes uncoupled from the vasculature in HDM exposed and vehicle treated animals. Pericytes were enumerated and the average number of cells per trachea was calculated. As expected, there was an increased number of uncoupled and migrating pericytes in the airways of HDM-exposed mice, but this number was significantly reduced in mice concurrently treated with LIT-927 (p < 0.05; Fig. 6C). A shape change was also evident in the pericytes of HDM + VEH mice, with a reduction in the area:perimeter ratio, with a trend toward longer, more myofibroblast-like cells under these conditions (p = 0.06; Fig. 6D). Further assessments of pulmonary pericytes by flow cytometry indicated a greater number of CXCR4 + pericytes obtained from the lungs of HDM-exposed mice compared to control mice (p < 0.05), but LIT-927 expression had no effect on the number (Fig. 5E) or proportion (Fig. 5F) of pericytes expressing CXCR4 + , nor on the expression level of CXCR4 on these cells (MFI; data not shown).

### LIT-927 abrogates pericyte migration and mitigates airway smooth muscle thickening

**Fig. 7:**
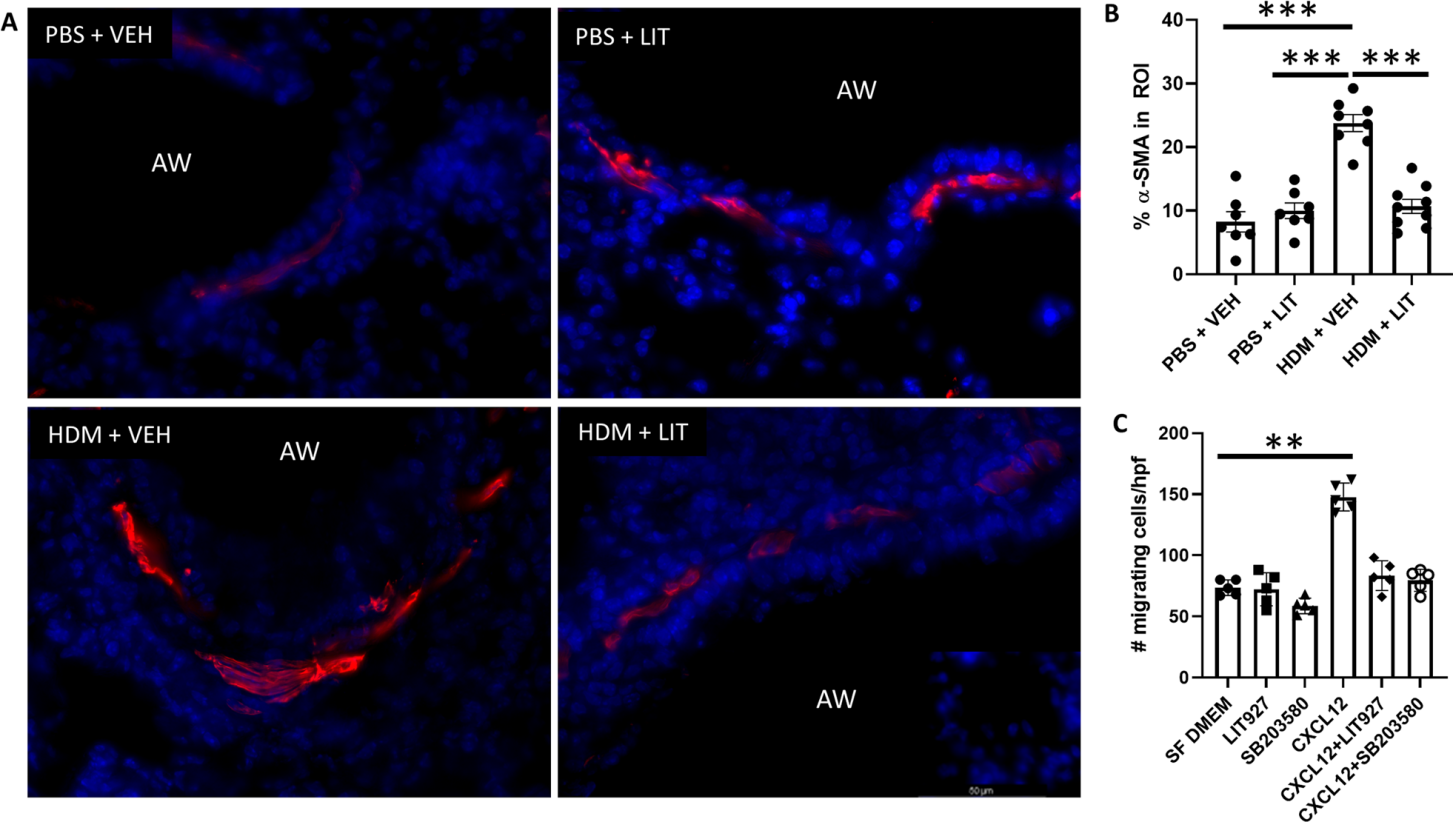
LIT-927 abrogates pericyte migration and mitigates airway smooth muscle thickening. Female C57/Bl6 mice (6–8 weeks old) were subjected to intranasal delivery of sterile PBS (10 µl) or house dust mite extract (HDM; 25 µg in 10 µl) 5 days a week for 5 consecutive weeks. **A** At the end of the protocol, lung sections obtained from PBS control and HDM-exposed mice were stained for α-smooth muscle actin (α-SMA; red) to assess the impact of LIT treatment on airway smooth muscle remodeling. Inset, negative control; scale bar 50 µm. **B** Airway smooth muscle thickness was quantified morphometrically using ImageJ software. n = 6–9 per group from two independent experiments; ***p < 0.001. Human placental pericytes were subjected to **C**. Transwell migration assays were used to assess cell migration to CXCL12 (300 ng/ml) in the absence or presence of the CXCL12 neutraligand LIT-927 and the p38 MAPK inhibitor SB203580. n = 5 per group, representative of three independent experiments; **p < 0.01. AW: airway; HDM: house dust mite; LIT: LIT-927 (CXCL12 neutraligand); PBS: phosphate-buffered saline; VEH: vehicle control

Immunofluorescent staining was performed on lung sections obtained from mice exposed to PBS/HDM for 5 weeks and treated with LIT-927/vehicle for the last 2 weeks of the experiment. Lung sections from these mice were stained for α-SMA to assess airway smooth muscle thickening (Fig. 7A). Increased accumulation of α-SMA+ cells was observed in the airway wall following HDM exposure; as expected, the airway smooth muscle layer was significantly thicker and more continuous in HDM-exposed mice treated with the vehicle Fig. 7A). In contrast, lungs from mice treated with LIT-927 in addition to HDM exposure had a thinner and more fragmented smooth muscle layer, similar to observations in the lungs of control mice (Fig. 7A). Quantification of the of α-SMA cells in the large airways revealed that LIT-927 treatment significantly reduced airway smooth muscle thickening in mice concurrently exposed to HDM (p < 0.001; Fig. 7B). Additional mechanistic studies revealed that HPLPC migration was abrogated by breaking the CXCL12 gradient using the CXCL12 neutraligand LIT-927 [18] as well as by inhibiting CXCR4 intracellular signalling using the p38 MAPK inhibitor SB203580 (p < 0.01; Fig. 7C, D).

## Discussion

In the context of tissue injury, inflammation, and fibrosis, pericytes have shown the capacity to differentiate into fibroblasts and contribute to wound healing [25]. Immunohistochemical analysis of dermal scars has suggested that a population of pericytes migrates into the perivascular space before developing into fibroblast-like cells [25]. More recently, studies have implicated pericytes as a rich source of myofibroblasts in mouse models of renal [9] and hepatic [8] fibrosis. Lin et al*.* used wild type mice chimeras bearing the bone marrow from mice expressing GFP-tagged collagen type 1α1 (*coll1a1*) to demonstrate that pericytes differentiate into myofibroblasts in a model of kidney fibrosis. Following ureteral obstruction, *coll1a1*-GFP-expressing pericytes were identified as the primary source of scar-forming myofibroblasts. Further studies by the same group in 2010 utilized Cre fate tracking techniques to show that FoxD1-positive and PDGFR-β cells (perivascular cells) proliferate and differentiate into alpha-smooth muscle actin (α-SMA) positive myofibroblasts during fibrosis. It was shown that these perivascular cells were responsible for > 95% of scar-forming myofibroblasts in this fibrotic model [26]. Mederacke et al*.* demonstrated, using Cre-transgenic mice, that liver-resident pericytes (hepatic stellate cells) are responsible for approximately 90% of tissue myofibroblasts in three models of liver fibrosis driven by toxin exposure, cholestasis and non-alcoholic steatohepatitis [8]. More recently, Johnson et al*.* delineated the role of PDGF-B/PDGFRβ signaling in maintaining pericyte-endothelial cell interactions in the HDM-driven mouse model of allergic lung disease. Pharmacological inhibition of PDGFRβ resulted in robust pericyte uncoupling from the vasculature, increased airway smooth muscle thickening and exacerbated lung dysfunction. This study was the first to demonstrate that pericytes migrate away from the tissue vasculature to subepithelial regions and contribute to airway remodeling by incorporating into smooth muscle bundles [10]. However, the mechanism driving pericyte migration to the airway wall was not identified.

As a first step in characterizing the phenotype and function of pulmonary pericytes in response to allergen exposure, we performed a screen of cell surface markers on freshly isolated lung pericytes. Consistent with the literature, pericytes lacked CD31, CD34, CD45, CD62L, CD71, CD106, c-kit and CD133 expression, and expressed CD49b, CD63, CD90, CD105 and CD146 [27]. Pulmonary pericytes were also found to be weakly positive for CD54 [28]. Moreover, according to the International Society of Cellular Therapy (ISCT) definition of human MSC [29], murine pulmonary pericytes can be defined as a subset of MSC based on their expression of CD105, CD73 and CD90, and the absence of CD45, CD34, CD11b, CD79a, CD19 and HLA-DR; however, pericytes were also CD14 positive. In the mouse, MSC express Sca1, which in conjunction with PDGFRα is used to define murine bone marrow MSC, known as PαS cells [30]. In this assay, pericytes showed moderate expression of Sca1 but little to no expression of PDGFRα. Other MSC markers including CD9, CD24, CD44, CD47, CD49e, CD81 and CD98, [31] were also identified on mouse pulmonary pericytes. Moreover, pericytes expressed the signaling receptor Notch2 and CD51, an integrin expressed on Nestin+/CD146+ pericyte-like MSCs [32].

Following this validation of a flow cytometry panel to identify murine pulmonary pericytes, mice were subjected to the HDM-driven model of chronic allergic inflammation to assess the impact of a Th2-polarized immune environment on pericyte phenotype and function. Compared to control mice, pericytes made up a smaller proportion of total lung cells, indicative of massive immune cell infiltration in this disease model. However, the expression levels of markers used to identify pericytes were unchanged, supporting the use of this technique to interrogate the role of pericytes in the development of airway remodeling. First, we used this pericyte labelling strategy to sort pericytes from the lung and assess their migratory capacity in the Transwell assay; indeed, pericytes from inflamed lungs migrated more readily to a chemotactic signal. Further assessments of marker expression on pericytes also demonstrated significant increases in CD13 and podoplanin, which have been previously shown to be implicated in mesenchymal cell migration [33, 34]. Moreover, based on previous studies demonstrating MSC migration in response to CXCL12 [16], we assessed the regulation of the CXCL12/CXCR4 pathway in HDM-exposed mice. Pericytes from the inflamed lung showed higher levels of CXCR4 than those from control mice, and the expression of its cognate ligand CXCL12 was significantly elevated in the bronchoalveolar lavage of mice exposed to HDM for a period of 5 weeks. Interestingly, CXCL12 expression remained at baseline after 3 weeks of HDM exposure; previous studies have shown that airway remodeling is not yet established at this time point and may not occur at all in milder cases of asthma [3, 35]. Furthermore, immunostained lung sections showed the accumulation of NG2+/CXCR4+ pericytes in the airway smooth muscle of HDM-exposed mice. In correlation with the murine pulmonary pericyte CXCR4 expression data, migration analysis using the Transwell assay also showed that human pulmonary pericytes exposed to TGF-β were more responsive to CXCL12, and that pericyte migration toward CXCL12 can be inhibited by interfering with CXCL12-CXCR4 interactions (LIT-927), to a similar degree as the inhibition induced by blocking CXCR4 intracellular signaling (SB203580).

Taken together, these results suggest that the CXCR4/CXCL12 axis may be functionally relevant in the chemotaxis of pericytes in this disease model. Although there is currently little knowledge of the relevance of this axis in pulmonary pericyte migration during inflammation, there is substantial literature concerning the role of the same axis in the recruitment of bone marrow mesenchymal stem cells in response to injury. Previous studies have shown that levels of CXCL12 transcript and protein expression are increased under hypoxic conditions, leading to the recruitment of circulating CXCR4 expressing progenitor cells to the site of injury, which participate in tissue regeneration. Similarly, levels of CXCL12 have been shown to increase following bone fracture [36]. Although these injuries are in a different context to the HDM model of inflammation used in this study, these findings strongly suggest that CXCL12 has a profound influence on pericyte/MSC recruitment in response to injury.

Previous studies have assessed the effect of CXCL12 on pericytes by using a CXCR4 inhibitor, AMD3100. This study showed that CXCL12 contributes to the differentiation of bone marrow stem cells into pericytes and the overall number of pericytes present at the site of the fibrotic tumor [37]. However, as AMD3100 has been shown to induce the egress of bone marrow MSC into the peripheral blood [17], in the context of allergic asthma, this effect could be detrimental. As an alternative strategy to mitigate these negative effects of AMD3100, recent studies have directly interfered with CXCL12 activity by developing a novel neutraligand molecule (LIT-927), which was modified from chalcone 4 in order to increase bioavailability [18]. The effect of LIT-927 has been shown in a mouse model of pulmonary hypertension by reducing pericyte migration toward blood vessels and mitigating the vascular remodeling characteristic of this disease [38]. In the context of HDM-driven allergic inflammation, we elected to deliver LIT-927 through the respiratory route (intranasally) in order to disrupt the CXCL12 gradient at the site of expression (inflammatory exudates in the airway wall). Moreover, we delivered this compound therapeutically, i.e. after the process of airway remodeling had been initiated but not yet fully established [3]. Using this strategy, we were able to observe that LIT-927 drastically reduced symptom scores in treated mice and led to a significant reduction in the airway smooth muscle burden. Mechanistically, this was found to be associated with a significant reduction in the number of pericytes uncoupling from the pulmonary vasculature, without an effect on CXCR4 expression levels or the number of pericytes expressing this chemokine receptor.

There are some limitations to our study. First, this study investigated the impact of LIT-927 treatment at a single concentration and duration of treatment; extensive dose- and time-response experiments are certainly warranted to more fully investigate the potential of CXCL12 neutralization as a therapy for allergic asthma. Furthermore, the lack of invasive measures of lung function in the present study did not allow us to fully interrogate the impact of LIT-927 treatment on lung function. Finally, the impact of LIT-927 on the influx of CXCR4+ inflammatory cells, most notably monocyte-derived macrophages, was not evaluated. However, a preliminary assessment of lung sections from PBS/HDM and VEH/LIT mice indicated little impact on the egress of these cells from the circulation. Combined with the absence of an impact of LIT-927 treatment on BAL cell numbers and differentials, these results suggest that intranasally delivered LIT-927 has only minor effects beyond the large airways.

In summary, the contribution of CXCR4+ pericytes to inflammation-driven tissue remodeling indicates a novel therapeutic target, i.e. CXCL12 neutralization, for the treatment of chronic allergic airway disease. We have presented evidence demonstrating the mechanism by which pulmonary pericytes migrate to the airway wall and contribute to the structural changes seen in persistent allergic airway inflammation, and we furthermore provide a viable pharmacological target for the mitigation of airway remodeling in allergic airway disease. Further investigations are needed using LIT-927 or other neutraligands of CXCL12 to determine the impact of long-term treatment and to explore the possibility of treatment in tandem with existing asthma therapies, such as corticosteroids.

## Data Availability

The data referring to this article (i.e. text, tables, and figures) will be made available upon reasonable request. Proposals should be directed to j.johnson1@aston.ac.uk.

## References

[1] AsthmaUK. Asthma facts and statistics. 2019; https://www.asthma.org.uk/about/media/facts-and-statistics/. Accessed 5 Feb 2019.

[2] Network GA. The Global Asthma Report 2018. 2018: Auckland, New Zealand.

[3] Johnson JR (2004). Continuous exposure to house dust mite elicits chronic airway inflammation and structural remodeling. Am J Respir Crit Care Med.

[4] Al-Muhsen S, Johnson JR, Hamid Q (2011). Remodeling in asthma. J Allergy Clin Immunol.

[5] Postma DS (2014). Asthma and chronic obstructive pulmonary disease: similarities and differences. Clin Chest Med.

[6] Barnes PJ (2012). Severe asthma: advances in current management and future therapy. J Allergy Clin Immunol.

[7] Chapman KR (2008). Suboptimal asthma control: prevalence, detection and consequences in general practice. Eur Respir J.

[8] Mederacke I (2013). Fate tracing reveals hepatic stellate cells as dominant contributors to liver fibrosis independent of its aetiology. Nat Commun.

[9] Lin SL (2008). Pericytes and perivascular fibroblasts are the primary source of collagen-producing cells in obstructive fibrosis of the kidney. Am J Pathol.

[10] Johnson JR (2015). Pericytes contribute to airway remodeling in a mouse model of chronic allergic asthma. Am J Physiol Lung Cell Mol Physiol.

[11] Feng J, Mantesso A, Sharpe PT (2010). Perivascular cells as mesenchymal stem cells. Expert Opin Biol Ther.

[12] Rowley JE, Johnson JR (2014). Pericytes in chronic lung disease. Int Arch Allergy Immunol.

[13] Wong SP (2015). Pericytes, mesenchymal stem cells and their contributions to tissue repair. Pharmacol Ther.

[14] Diaz-Flores L (2009). Pericytes. Morphofunction, interactions and pathology in a quiescent and activated mesenchymal cell niche. Histol Histopathol.

[15] Rankin SM (2012). Chemokines and adult bone marrow stem cells. Immunol Lett.

[16] Hu C (2013). CXCL12/CXCR4 axis promotes mesenchymal stem cell mobilization to burn wounds and contributes to wound repair. J Surg Res.

[17] Kumar S, Ponnazhagan S (2012). Mobilization of bone marrow mesenchymal stem cells in vivo augments bone healing in a mouse model of segmental bone defect. Bone.

[18] Regenass P (2018). Discovery of a locally and orally active CXCL12 Neutraligand (LIT-927) with anti-inflammatory effect in a murine model of allergic airway hypereosinophilia. J Med Chem.

[19] du Sert NP (2020). The ARRIVE guidelines 2.0: updated guidelines for reporting animal research. J Physiol.

[20] Sali A, Blundell TL (1993). Comparative protein modelling by satisfaction of spatial restraints. J Mol Biol.

[21] Barth P, Schonbrun J, Baker D (2007). Toward high-resolution prediction and design of transmembrane helical protein structures. Proc Natl Acad Sci U S A.

[22] Morris GM (2009). AutoDock4 and AutoDockTools4: automated docking with selective receptor flexibility. J Comput Chem.

[23] Vakser IA, Matar OG, Lam CF (1999). A systematic study of low-resolution recognition in protein–protein complexes. Proc Natl Acad Sci USA.

[24] Castrechini NM (2010). Mesenchymal stem cells in human placental chorionic villi reside in a vascular Niche. Placenta.

[25] Sundberg C (2002). Stable expression of angiopoietin-1 and other markers by cultured pericytes: phenotypic similarities to a subpopulation of cells in maturing vessels during later stages of angiogenesis in vivo. Lab Invest.

[26] Humphreys BD (2010). Fate tracing reveals the pericyte and not epithelial origin of myofibroblasts in kidney fibrosis. Am J Pathol.

[27] Dellavalle A (2007). Pericytes of human skeletal muscle are myogenic precursors distinct from satellite cells. Nat Cell Biol.

[28] Turley SJ, Cremasco V, Astarita JL (2015). Immunological hallmarks of stromal cells in the tumour microenvironment. Nat Rev Immunol.

[29] Dominici M (2006). Minimal criteria for defining multipotent mesenchymal stromal cells. The International Society for Cellular Therapy position statement. Cytotherapy.

[30] Morikawa S (2009). Prospective identification, isolation, and systemic transplantation of multipotent mesenchymal stem cells in murine bone marrow. J Exp Med.

[31] Rostovskaya M, Anastassiadis K (2012). Differential expression of surface markers in mouse bone marrow mesenchymal stromal cell subpopulations with distinct lineage commitment. PLoS ONE.

[32] Pinho S (2013). PDGFR alpha and CD51 mark human Nestin(+) sphere-forming mesenchymal stem cells capable of hematopoietic progenitor cell expansion. J Exp Med.

[33] Rahman MM (2014). CD13 promotes mesenchymal stem cell-mediated regeneration of ischemic muscle. Front Physiol.

[34] Ward L (2019). Podoplanin regulates the migration of mesenchymal stromal cells and their interaction with platelets. Ann Rheum Dis.

[35] Elliot JG (2015). Distribution of airway smooth muscle remodelling in asthma: relation to airway inflammation. Respirology.

[36] Ceradini DJ (2004). Progenitor cell trafficking is regulated by hypoxic gradients through HIF-1 induction of SDF-1. Nat Med.

[37] Hamdan R, Zhou ZC, Kleinerman ES (2014). Blocking SDF-1 alpha/CXCR4 downregulates PDGF-B and inhibits bone marrow-derived pericyte differentiation and tumor vascular expansion in Ewing tumors. Mol Cancer Ther.

[38] Bordenave J (2019). Neutralization of CXCL12 attenuates established pulmonary hypertension in rats. Cardiovasc Res.

